# Increased Incidence of Urinary Tract Infections in Newborns With Neonatal Opiate Withdrawal Syndrome Treated With Morphine and Clonidine: A Case Series With Supplemental Descriptive Cohort Data

**DOI:** 10.1155/crpe/8825061

**Published:** 2026-07-18

**Authors:** Alexandra A. Richmond, M. Cody Smith, Micah Butcher, Lesley Cottrell, Mark J. Polak

**Affiliations:** ^1^ Department of Medical Education, West Virginia University School of Medicine, Morgantown, West Virginia, USA, wvu.edu; ^2^ Department of Pediatrics, Section of Neonatology, West Virginia University School of Medicine, WVU Medicine Golisano Children’s Hospital, Morgantown, West Virginia, USA, wvu.edu; ^3^ Department of Pharmacy Services, WVU Medicine Golisano Children’s Hospital, Morgantown, West Virginia, USA; ^4^ Department of Pediatrics, West Virginia University School of Medicine, Morgantown, West Virginia, USA, wvu.edu

**Keywords:** clonidine, neonatal opioid withdrawal syndrome, urinary tract infection

## Abstract

Neonatal opioid withdrawal syndrome has become a prevalent diagnosis with the rise of maternal substance use during pregnancy. Manifestation of this syndrome is characterized by altered activity of the central nervous system with symptoms including irritability, gastrointestinal dysfunction, and activation of the autonomic nervous system. There is currently no standardized pharmacological protocol for the treatment of this syndrome. Opioids such as morphine and methadone are first‐line medications with clonidine as the recommended adjunct therapy. We observed increases in urinary tract infection in infants, especially male infants, with neonatal opioid withdrawal syndrome that were treated with morphine and clonidine. Urinary tract infection is uncommon among newborns and poses a significant risk to their health secondary to their immature renal and immune systems. We describe 4 cases in newborns treated with morphine and clonidine, prompting an investigation into the individual cases to highlight a potential association between clonidine exposure and urinary tract infection.

**Information Box**
- What specific question(s) does this report address?
- Is there an association between clonidine treatment and urinary tract infection (UTI) in infants diagnosed with neonatal opioid withdrawal syndrome?
- What does this report add to our current knowledge?
- Incidence of UTI may be increased in patients with neonatal opioid withdrawal syndrome who are treated with adjunctive clonidine.

## 1. Introduction

Neonatal opioid withdrawal syndrome (NOWS) is a manifestation of withdrawal symptoms in infants exposed to intrauterine opioids. With opioid use continuing to increase, NOWS has become a significant diagnosis in terms of prevalence and study [1].

Treatment of NOWS is focused on treating symptoms associated with increased noradrenergic activity [1], which includes hypertonia, tremors, exaggerated reflexes, irritability, fever, and diarrhea [2]. It has become standard practice to progress to pharmacological means when nonpharmacologic measures fail. Opioids, such as morphine, are considered first‐line treatments, with clonidine being a common adjunctive therapy. Clonidine, an Alpha‐2 receptor agonist, reduces excessive noradrenergic activity and does not carry as deleterious neurotoxic effects as phenobarbital, an agent commonly used in the treatment of NOWS [3]. However, it has been associated with urinary retention and altered urinary tract function [4, 5].

Our Neonatal Intensive Care Unit uses a standardized guideline utilizing the Modified Finnegan Neonatal Abstinence Score (MFNAS) to identify infants with NOWS that require pharmacotherapy. We use morphine as first‐line NOWS treatment starting with symptom‐based dosing and advancing to scheduled morphine taper if neonates require 3 doses within a 24‐h period. We start clonidine as adjuvant treatment when symptoms fail to respond to escalating doses of scheduled morphine [6].

UTIs may predispose patients to the development of severe complications, including bacteremia, sepsis, and renal scarring [7]. Additionally, exposure to antibiotics predisposes to subsequent extended spectrum beta‐lactamase (ESBL) producing bacteria, further highlighting the importance of preventing UTI in this population [8].

The incidence of neonatal UTI is not clearly established, and due to nonspecific symptoms, it can be difficult to detect in this age group [9]. Considering the severity of the consequences of UTI in neonates, they must be diagnosed and treated promptly.

We describe 4 cases of UTI in infants diagnosed with NOWS who were treated with clonidine as an adjunct medication to morphine. We compared these patients to those managed nonpharmacologically or with morphine alone. We also included an equal number of babies with no exposures. They were matched manually in terms of year of birth, sex, and gestational age. Due to the relatively low prevalence of UTI in neonates, the potential association of UTI with the combination of morphine and clonidine may be significant and guide treatment measures when infants with NOWS require clonidine.

This project was approved by the Institutional Review board (IRB) by our institution as an expedited chart review project (# 2403947159). Consents for inclusion were not required.

### 1.1. Statistical Analysis

This study used a retrospective observational design comparing infants without NOWS, infants with NOWS managed without clonidine, and infants with NOWS treated with morphine and clonidine, including a subgroup who developed UTI. Descriptive statistics were used to summarize infant and maternal characteristics. Continuous variables are presented as means with standard deviations (SD) when approximately normally distributed and as medians with interquartile ranges (IQRs) when distributions were skewed. Categorical variables are reported as counts and percentages.

Comparisons between groups were performed using Student’s *t*‐tests or one‐way analysis of variance (ANOVA) for continuous variables with approximate normality. When assumptions of normality were not met, nonparametric tests (Mann–Whitney U or Kruskal–Wallis tests) were used as appropriate. Chi‐square tests or Fisher’s exact tests were applied for comparisons of categorical variables.

All statistical tests were two‐sided, and a *p* value of < 0.05 was considered statistically significant. *p* values are reported in Tables 1 and 2 to indicate unadjusted comparisons across groups. Missing data were minimal and occurred primarily due to incomplete documentation in the electronic medical record. Analyses were conducted using complete‐case analysis, with denominators adjusted accordingly for variables with missing values. No imputation procedures were performed. All statistical analyses were conducted using SPSS Version 31.0. Statistical analysis was not applied when cell counts were less than five.

**TABLE 1:** Infant birth and treatment characteristics.

Infant data	Infants w/o NOWS (*n* = 66)	Infants w/NOWS (*n* = 66)	NOWS + clondine (*n* = 60)	NOWS + clonidine + UTI (*n* = 4)	*p* < 0.05
Gestation (weeks)	37 ± 3	38 ± 2	39 ± 2	40 ± 1	NS
Inborn	100%	76%	48%	50%	NS
Male	53%	58%	52%	83%	NS
C‐section	44%	39%	30%	17%	NS
Instrumented vaginal deliveries	3%	3%	5%	0^∗^	< 0.05
Length of stay (days)	14 ± 43	13 ± 8	27 ± 11^∗^	33 ± 14^∗^	< 0.01
Apgar 1 min	7.6	7.7	7.1	7.8	NS
Apgar 5 min	8.6	8.5	8.4	8.7	NS
Z score for weight	−0.06 ± 0.90	−0.61 ± 1.08^∗^	−0.67 ± 0.91^∗^	−0.83 ± 0.66^∗^	< 0.001
Large for gestational age (LGA)	5%	3%	3%	0^∗^	< 0.005
Small for gestational age (SGA)	5%	29%^∗^	22%^∗^	33%^∗^	< 0.01
Twin	9%	8%	0%	0	NS
Congenital anomaly	8%	5%	12%	17%	NS
Microcephaly (< 3% for gestational age)	0%	8%	10%	0	NS
NOWS	0%	100%^∗^	100%^∗^	100%^∗^	< 0.001
Rescue morphine		50%	40%	50%	NS
Morphine taper		52%	100%	100%	NS
Morphine taper start (avg day)		4 ± 2	3 ± 2	3 ± 3	NS
Clonidine		—	100%^†^	100%^†^	< 0.001
Clonidine start (avg day)		—	8 ± 6	11 ± 9	NS
UTI		1.5%	0%	100%^∗∗^	< 0.001
UTI day (avg)		20	—	25 ± 8	NS
Breast milk		27%	12%	17%	NS
Formula		94%	97%	100%	NS
Hydrolyzed formula		53%	67%	83%	NS

*Note:* All infants were born between 2018 and 2024. Infants without NOWS were matched with the same time periods as NOWS infants.

^∗^Significant difference in all NOWS groups compared to non‐NOWS infants.

^†^Significant difference in NOWS infants needing clonidine versus NOWS infants without clonidine.

^∗∗^Significance in NOWS infants needing clonidine with UTI compared to all other NOWS infants.

**TABLE 2:** Maternal characteristics, diagnoses, and substance use.

Maternal data	Maternal data from 4‐week period 2023 (*n* = 284)	Maternal data for infants with NOWS (2018–2023) (*n* = 60)	Maternal data for infants with NOWS Morphine + Clonidine (2018–2023) (*n* = 66)	*p* < 0.05
Maternal age (years)	28 ± 5	29 ± 5	30 ± 4	NS
Survival	100%	100%	100%	NS
BMI (kg/m^2^)	34.57 ± 8.45	29.66 ± 18.0	29.30 ± 0.44	NS
BMI > 30	69%	39%	44%	NS
Hepatitis C positive	4%	40%^∗^	45%^∗^	< 0.01
Hypertension (+ preeclampsia & HELLP^†^)	38%	17%	13%	NS
Diabetes (GDM^∗^, Type 1 and Type 2)	11%	0^∗^	0^∗^	< 0.05
Prenatal care (at least 1 visit)		89%	83%	NS
Drug treatment		67%	78%	NS
Tobacco	15%	83%^∗^	70%^∗^	< 0.01
Marijuana	7%	19%	17%	NS
Opioid exposure	4%	100%^∗^	100%^∗^	< 0.01
Methadone		18^†^	34%^†^	< 0.05
Buprenorphine		40%	36%	NS
Buprenorphine‐naloxone		31%	17%	NS
Heroin		22%	33%	NS
Fentanyl		17%	19%	NS
OxyContin/oxycodone		3%	3%	NS
Benzodiazepine		12%	13%	NS
Methamphetamine	2%	25%^∗^	28%^∗^	< 0.05
Cocaine	0	19%^∗^	26%^∗^	< 0.05
Gabapentin		7%	6%	NS
Inhalants		3%	2%	

*Note:* GD: gestational diabetes mellitus. Except for use of methadone (^†^all NOWS compared to NOWS needing morphine & clonidine), all significant differences (^∗^) were between the 2023 cohort and both NOWS groups.

^‡^HELLP: hemolysis, elevated liver enzymes, and low platelets.

### 1.2. Case Series

Tables 1 and 2 describe newborns and their mothers for 4 groups, newborns born to mothers with no exposures, newborns with NOWS treated nonpharmacologically or with morphine, newborns with NOWS treated with morphine and clonidine, and the 4 infants with NOWS who developed UTI while treated for NOWS with morphine and clonidine.

**TABLE 3:** Baseline labs.

	Hemoglobin	Hematocrit	Bilirubin, total	Bilirubin, conjugated	Glucose, POC
Patient A	17.7	52.0	14.1	0.6	59
Patient B	12.5	34.5			128
Patient C	14.4	40.5			128
Patient D	17.9	52.3		79	126

Patient A was a white male outborn at 40w3d gestational age via SVD to a G1P1 28‐year‐old mother diagnosed with hepatitis C. Intrauterine substance exposure consisted of buprenorphine‐naloxone and a selective serotonin reuptake inhibitor (SSRI). Prenatal care was adequate. The infant was diagnosed with NOWS. Patient B required 3 doses of morphine on DOL 1 (0.05 mg/kg) followed by initiation of scheduled morphine taper (highest dose 0.15 mg/kg every 3 h) beginning DOL 2 and ending on DOL 22. Clonidine was initiated on DOL 5 (highest dose 1.5 mcg/kg every 3 h) due to an acute increase in MFNAS scores and ended on DOL 25. On DOL 16, the infant presented with bloody stools, prompting infections workup with blood and urine (via straight catheter). Blood culture revealed no growth after 24 h. Urine culture grew *E. coli*, and one‐time treatment with IM ceftazidime (50 mg/kg) was initiated followed by transition to oral amoxicillin (30 mg/kg/day divided twice a day) for a 10‐day course. Renal ultrasound was normal on DOL 26. He was discharged on DOL 26 with a LOS of 26 days.

Patient B was a white male outborn at 38w4d gestational age via vaginal birth after cesarean (VBAC) to a G3P2 25‐year‐old mother. Maternal information was unavailable due to absent prenatal care, but at time of delivery, intrauterine substance exposure consisted of buprenorphine, oxycodone, gabapentin, tetrahydrocannabinol (THC), benzodiazepines, amphetamines, and methamphetamines. Alcohol use was suspected. The infant was diagnosed with NOWS and required morphine through DOL 32 (highest dose 0.125 mg/kg every 3 h). Clonidine taper was started on DOL 15 (highest dose 1 mcg/kg every 3 h) due to an acute increase in MFNAS scores despite treatment with morphine, ending on DOL 32. On DOL 26, the infant presented with a fever of 100.8° F, prompting an infections workup with blood and urine cultures as well as respiratory viral panel. He was positive for respiratory syncytial virus and developed respiratory distress. Blood culture was negative. Urine culture grew *E. coli*, and he was treated with IV ampicillin (100 mg/kg divided every 8 h for 3 days and 50 mg/kg every 8 h for 4 days) for a total of 7 days. A subsequent renal ultrasound showed Grade 1 hydronephrosis, prompting initiation of daily amoxicillin prophylaxis with referral to Pediatric Nephrology as an outpatient. At 57 days old, ultrasound revealed improvement of hydronephrosis. Approximately 5 months after discharge, voiding cystourethrogram revealed the absence of vesicoureteral reflux, and the patient was instructed to discontinue prophylaxis antibiotics. He was discharged on DOL 34 with a LOS of 19 days at our institution after initial treatment at an outlying facility for 15 days.

Patient C was a white female inborn at 40w3d gestational age via SVD to a G6P4 25‐year‐old mother that had adequate prenatal care. Intrauterine substance exposure consisted of buprenorphine and phenobarbital. Tobacco use was suspected. The infant was diagnosed with NOWS. Patient C required initiation of morphine (with a highest dose of 0.225 mg/kg every 3 h) beginning DOL 2 and ending on DOL 39 with several additional rescue doses (0.025–0.05 mg/kg). Clonidine taper was subsequently begun on DOL 3 (with a highest dose of 3 mcg/kg every 6 h) due to an acute increase in MFNAS scores despite treatment with increasing doses of morphine, ending on DOL 48. Patient D’s course did not follow typical withdrawal patterns. Upon suspicion that she was also withdrawing from benzodiazepines, lorazepam was initiated on DOL 12 with immediate clinical improvement (0.1 mg/kg). Lorazepam was subsequently administered on a schedule (with a highest dose of 0.1 mg/kg every 6 h). She required 41 total days of lorazepam. Beginning DOL 25, phenobarbital (with a highest dose of 5 mg/kg/day divided twice a day) was initiated for further management of extreme irritability secondary to NAS, ending on DOL 44. On DOL 36, the infant presented with fever up to 102.4°F and bloody stool, prompting full infectious evaluation. Significant laboratory findings included atypical lymphocytes and elevated C‐reactive protein of 137.7 mg/L. Cerebrospinal fluid cell count was within normal limits. Urine culture grew *E. coli*, and empiric treatment with IV vancomycin and gentamicin was initiated, then deescalated to IV ampicillin (200 mg/kg/day divided every 6 h) for a 10‐day course. Renal US showed no evidence of hydronephrosis or pyelonephritis. She was discharged on DOL 57 with a LOS of 57 days.

Patient D was a white male inborn at 42w4d gestational age via cesarean to a G3P2 25‐year‐old mother. Intrauterine substance exposure consisted of buprenorphine, heroin, nicotine, and amphetamines. Prenatal care was limited. The infant was diagnosed with NOWS. Patient E required morphine beginning DOL 2 and ending on DOL 25 (with a highest dose of 0.125 mg/kg every 3 h) with several intermittent rescue doses (0.025–0.05 mg/kg). Clonidine taper was subsequently begun on DOL 2 due to an acute increase in MFNAS scores despite treatment with morphine, ending on DOL 29 (with a highest dose of 2.5 mcg/kg every 6 h). On DOL 22, the infant was noted to be lethargic and pale, prompting collection of urine for urinalysis and culture as well as blood cultures. Blood cultures were negative. Urine culture grew *Klebsiella*, and coagulase‐negative *Staphylococcus* and treatment regimen consisted of 4 days of IV ceftazidime (50 mg/kg 3 times a day) and 3 days oral amoxicillin‐clavulanate (37 mg/kg/day). Renal ultrasound had evidence of left Grade 1 hydronephrosis. The infant was discharged on DOL 29 with a LOS of 29 days. A voiding cystourethrogram performed at 5 months of age was normal.

Table 1 details breakdown of timing of UTI in relation to the development of NOWS and therapy instituted and the bacteria that grew from the catheterized urine specimens. The baseline laboratory results on the day that UTI was diagnosed for the four patients are detailed in Table 3.

## 2. Discussion

Prenatal substance exposure is a significant issue for maternal and neonatal care in our region of Appalachia. Opioids, such as morphine and methadone, have been the first‐line medications for NOWS for decades. Second‐line medications are initiated when symptoms persist despite opioid treatment. Currently, clonidine is the recommended adjunctive treatment [6]. The four infants presented represent 6% of infants with severe NOWS treated with morphine and clonidine, compared to a 1% incidence of UTI in infants with NOWS at our institution, which is a similar incidence for all infants [10]. Overall, UTI is an uncommon diagnosis in infants, and while a cohort of four patients is limited, the difference in UTI incidence between groups at our institution brings attention to a potential relationship between clonidine and UTI which has not previously been reported. Invasive assessments for infection (blood cultures and catheterized urine cultures) are not part of standard practice for infants with NOWS. Clinically, these infants presented with acute worsening of NOWS symptoms along with typical signs and symptoms of infection (lethargy, fever, and bloody stools) when they had previously been tolerating scheduled weaning of NOWS medications.

Morphine and clonidine have synergistic analgesic effects [11]. There is also potential for additive adverse effects. A review by Verhamme et al. suggested that up to 10% of episodes of urinary retention may be associated with concomitant medication use [4]. Clonidine has been shown to alter urethral resistance and bladder function through effects on alpha receptors in the smooth muscle of the upper and lower urinary tracts [6]. Urinary retention with the use of clonidine has been reported, but the frequency is not defined [12], while the effects of opioids, including morphine, on renal function are widely known [13]. An increase in the incidence of urinary retention with the possibility of UTI should be considered when using clonidine in combination with morphine.

UTIs are associated with significant sequelae in infants, including bacteremia, meningitis, and renal scarring. According to Thomson et al., 6%–10% of infants with UTIs have concomitant bacteremia, which can lead to meningitis [14]. The risk was greater in infants aged 0–29 days, suggesting a potential relationship between age and meningitis risk. Furthermore, UTI may progress to acute pyelonephritis, which can cause renal scarring and subsequent hypertension, preeclampsia, and end‐stage renal disease later in life in up to 15% of children with first UTI [15]. Finally, recent antibiotic use and hospitalization can lead to the development of UTI caused by ESBL‐producing bacteria [8]. These long‐term consequences highlight the need for awareness surrounding UTI in higher‐risk infants to increase prevention efforts. Patient D was noted to have Grade 1 hydronephrosis which likely increased the likelihood of developing a UTI.

Regarding sex differences, males comprise 70%–90% of neonatal UTI cases [9]. This has been linked to the absence of circumcision as well as the greater incidence of structural abnormalities in male infants [16]. Additionally, male infants have been shown to have greater residual volumes and lower voiding pressures than females [9]. Our study population aligns with these statistics, with five out of six patients with UTI being male. Therefore, providers of patients with NOWS should be aware of the risk factors for UTI development in the first month of life and ensure that prevention and detection are a priority.

## 3. Conclusion

Our presentation is based on a retrospective review, although in our practice, infants who require clonidine as an adjuvant are monitored more closely, and a urine culture is considered if there are unexpected changes in the expected trajectory in the treatment course. Infants who require multiple medications to control NOWS symptoms do not follow a regular path toward resolution, and it may be difficult to discern whether a sudden increase in MFNAS scores is a worsening of NOWS or something else. The 4 infants we present all had signs and symptoms of infection (fever, a change in activity, lethargy, and blood in the stools). Even though our conclusions have limitations, our findings suggest that there may be an increased risk of UTI in infants, especially male infants, receiving morphine and clonidine for NOWS treatment. Neonatal providers should be mindful of the increased risk of UTIs in this patient group, especially if NOWS symptoms worsen in a neonate previously tolerating a scheduled weaning plan.

## Funding

No funding was received for this manuscript.

## Conflicts of Interest

The authors declare no conflicts of interest.

## Data Availability

The data that support the findings of this study are available on request from the corresponding author. The data are not publicly available due to privacy or ethical restrictions.
